# Atrial septal defect closure in a young patient presenting with recurrent cryptogenic stroke: a case report

**DOI:** 10.1186/s13019-020-01220-0

**Published:** 2020-07-25

**Authors:** Rakan I. Nazer

**Affiliations:** Department of Cardiac Science, King Fahad Cardiac Center, College of Medicine, King Saud University, Riyadh, 12372-7143 Kingdom of Saudi Arabia

**Keywords:** Stroke, ASD

## Abstract

**Background:**

Having an inter-atrial shunt in the form of a patent foramen ovale or atrial septal defect increases the risk of developing cryptogenic stroke. Prompt action is required in order to prevent stroke recurrence. The source of embolization may not be clear on stroke workup.

**Case presentation:**

A young female acutely presented with recurrent embolizations to the eye and brain. She was found to have an atrial septal defect. No clear intra-cardiac source of embolization was detected on workup including trans-esophageal echocardiography. Given the options between surgical versus device closure, the attending team opted for the surgical closure which yielded on direct left heart inspection small organized clots adherent to the tips of the mitral valve leaflets.

**Conclusions:**

The case report illustrates the potential advantages of the direct surgical closure in detecting and extracting the embolization source in patients who present with recurrent cryptogenic stroke.

## Background

Cryptogenic stroke is defined as cerebral or retinal infarct without a clear source of embolization or thrombosis. This type of stroke accounts for 30 to 40% of all ischemic strokes [1]. Having a persistent shunt between the left and right atrium in the form of a patent foramen ovale (PFO) or atrial septal defects (ASD) is associated with an increased risk for cryptogenic strokes in patients younger than 55 years of age [2]. In the absence of any other cardiac source of embolization in patients with interatrial shunts, cardioembolic strokes can happen by either “paradoxic” embolization of a venous-based clot travelling through the shunt, or by developing atrial fibrillation as a consequence of left atrial remodeling and blood stasis in the left atrial appendage [3].

In the following report, we summarize a case of a young female who first presented recurrent embolization in the eye and brain and was found to have a small fenestrated ASD. A closer direct intra-cardiac examination of the left heart during the surgical closure revealed the source which was not detected on cardiac imaging prior to surgery.

## Case presentation

A 22-year-old female is on chronic remission treatment for ulcerative colitis, initially presented acutely after experiencing a sudden loss of vision in the right eye. This was found to be secondary to an acute occlusion of the retinal artery. During her hospitalization, she experienced a sudden weakness in the left side of her body. The weakness gradually resolved over 72 h of onset. The patient was evaluated by the attending medical services and was diagnosed to have a cryptogenic stroke with recurrent embolization. She was initiated on low dose aspirin and the novel oral anticoagulant rivaroxaban.

As part of the screening of the embolization source, the patient had a magnetic resonance angiography of the brain which revealed nonspecific bilateral periventricular and subcortical white matter hyperintense foci. Ultrasound Doppler of both carotid arteries was negative. The screening for hematologic hypercoagulable conditions, autoimmune disease, and heparin induced thrombocytopenia was also negative. The holter ECG surveillance showed no evidence of arrhythmia. The tras-thoracic echocardiography revealed a small PFO with a restricted shunt from the left to the right side. Further cardiac evaluation by trans-esophageal echocardiography yielded a small fenestrated secondum ASD (0.8 cm × 1.2 cm) associated with mild right ventricular volume overload. The rest of the cardiac imaging was non-significant. Both the mitral and aortic valves were normal in structure and function with no evident clots in the left atrial appendage.

After addressing the patient’s condition in the combined interventional cardiology and cardiac surgery meeting, it was decided that the patient would be better served by a surgical closure of the ASD as opposed to a device closure. The fenestrated ASD and the inability to conclusively exclude a possible embolization source within the heart by imaging were strong points for the surgical closure. The heart was approached via a median sternotomy. Cardio-pulmonary bypass was initiated through direct aortic and bi-caval cannulation. The heart was arrested with blood cardioplegia and the right atrium was opened. The fenestrated ASD and the floppy rims were conglomerated in one clean defect. Further inspection of the left heart through the defect was seemingly insignificant except when the cooptation margins of the P2/A2 scallops of the mitral valve leaflets were pulled in view from the left ventricular cavity. This unexpectedly revealed two discrete masses (0.3 cm × 0.2 cm × 0.2 cm) which were adherent to the margins of the P2/A2 scallops. They were easily picked-out using a tissue forceps (see supplementary video). The site of the extracted mass from the margins of the mitral leaflets left a central mitral regurgitation jet which was negated by placing an annuoplasty band.

Post-operatively, the patient made a quick recovery. Her post-operative echocardiographic study showed no residual shunting and a normally functioning mitral valve. She was discharged home on aspirin and rivaroxabam for 3 months, then to continue treatment with low dose aspirin indefinitely. Both the histopathology and culture of the specimens were negative for any organisms or growth. The findings were consistent with a mature clot formation. The patient continues to do well on all subsequent clinic visits.

## Discussion and conclusions

Cryptogenic stroke is a devastating condition which usually affects younger groups without obvious atherogenic risk factors. It is estimated that 40% of patients with cryptogenic stroke will have some form of inter-atrial shunting and the risk of stroke seems independent of the shunt size [4]. Secondary stroke prevention is the cornerstone of management. Current evidence favor prompt intervention with percutaneous or surgical closure of the shunt to reduce the risk of recurrent strokes, yet it does not eliminate stroke risks [3]. This is due to the difficulty in estimating how much of the stoke burden is attributed solely to the shunt. The described case, stresses the need for an individualized assessment of each patient and potentially favors the direct surgical closure in those who present with recurrent embolization and ASDs. This entails the identification of shunts with high-risk morphological features and the utilization of the RoPE score to determine the probability of the shunt being responsible for causing the stroke [5]. Surgical closure may offer an extra advantage over device closure by allowing the direct inspection and extraction of a possible embolization source within the left heart not picked-up on preoperative imaging. This should be followed by vigilant rhythm monitoring and the consideration for anticoagulation.

## Supplementary information

**Additional file 1.**

## Data Availability

Available.

## Abbreviations

ASD: Atrial septal defect
PFO: Patent foramen ovale
